# Mesenchymal Stem Cells Expressing Brain-Derived Neurotrophic Factor Enhance Endogenous Neurogenesis in an Ischemic Stroke Model

**DOI:** 10.1155/2014/129145

**Published:** 2014-02-05

**Authors:** Chang Hyun Jeong, Seong Muk Kim, Jung Yeon Lim, Chung Heon Ryu, Jin Ae Jun, Sin-Soo Jeun

**Affiliations:** ^1^Department of Biomedical Science, College of Medicine, The Catholic University of Korea, Seoul 137-701, Republic of Korea; ^2^Postech-Catholic Biomedical Engineering Institute, The Catholic University of Korea, Seoul 137-701, Republic of Korea; ^3^Department of Neurosurgery, Seoul St. Mary's Hospital, The Catholic University of Korea, Seoul 137-701, Republic of Korea

## Abstract

Numerous studies have reported that mesenchymal stem cells (MSCs) can ameliorate neurological deficits in ischemic stroke models. Among the various hypotheses that have been suggested to explain the therapeutic mechanism underlying these observations, neurogenesis is thought to be critical. To enhance the therapeutic benefits of human bone marrow-derived MSCs (hBM-MSCs), we efficiently modified hBM-MSCs by introduction of the brain-derived neurotrophic factor (*BDNF*) gene via adenoviral transduction mediated by cell-permeable peptides and investigated whether *BDNF*-modified hBM-MSCs (MSCs-BDNF) contributed to functional recovery and endogenous neurogenesis in a rat model of middle cerebral artery occlusion (MCAO). Transplantation of MSCs induced the proliferation of 5-bromo-2′-deoxyuridine (BrdU-) positive cells in the subventricular zone. Transplantation of MSCs-BDNF enhanced the proliferation of endogenous neural stem cells more significantly, while suppressing cell death. Newborn cells differentiated into doublecortin (DCX-) positive neuroblasts and Neuronal Nuclei (NeuN-) positive mature neurons in the subventricular zone and ischemic boundary at higher rates in animals with MSCs-BDNF compared with treatment using solely phosphate buffered saline (PBS) or MSCs. Triphenyltetrazolium chloride staining and behavioral analysis revealed greater functional recovery in animals with MSCs-BDNF compared with the other groups. MSCs-BDNF exhibited effective therapeutic potential by protecting cell from apoptotic death and enhancing endogenous neurogenesis.

## 1. Introduction

Mesenchymal stem cells (MSCs) have displayed capabilities in regenerative therapy for the treatment of stroke, as a ready supply of angiogenic, antiapoptosis, and mitogenic factors and for their ability to migrate in damaged tissue and differentiate into neuronal cells [1, 2]. MSCs possess and can secrete neurotrophic factors [2, 3]. Transplantation of MSCs enhances functional recovery and endogenous neurogenesis in stroke models [4–6].

Endogenous neural stem cells (NSCs) have been identified in the brain and spinal cord of embryonic, postnatal, and adult rodents and primates [7, 8]. These cells reside mainly in the subventricular zone (SVZ) and in the subgranular zone (SGZ) of the hippocampus [9, 10]. When adult rodents are subjected to middle cerebral artery occlusion (MCAO), the resulting infarction injury stimulates a low level of endogenous neurogenesis in the SVZ of the affected side [11]. This is a promising target for therapeutic intervention, as it may be amenable to enhancement by adding appropriate factors that increase proliferation, migration, and differentiation, as well as functional integration [11]. Some candidate factors, including epidermal growth factor (EGF), fibroblast growth factor-2 (FGF-2), brain-derived neurotrophic factor (BDNF), granulocyte-colony stimulating factor (G-CSF), vascular endothelial growth factor (VEGF), glial cell-derived neurotrophic factor (GDNF), and insulin-like growth factor-1 (IGF-1), enhance the endogenous response to acute and subacute ischemic stroke [11–18].

BDNF is secreted by brain cells and induces neuroprotection. BDNF also crucially promotes the synaptic and axonal plasticity associated with learning, memory, and sensorimotor recovery [15]. As BDNF can stimulate neuronal differentiation in vitro, it has also been used to induce neurogenesis after focal ischemia [15, 19]. In addition to qualitatively enhancing neural structural plasticity, BDNF increases the number of newborn neurons in several regions of the brain [15]. Administration of *BDNF* modified MSCs has produced therapeutic benefits in a rat model of transient MCAO [20]. However, the potential of this approach regarding endogenous neurogenesis is unclear.

To test the hypothesis that a combined therapy of BDNF and MSCs enhances endogenous neurogenesis to a greater extent than MSCs alone, the present study investigated the effects of intracranial transplantation of *BDNF* modified human bone marrow-derived MSCs (MSCs-BDNF) on functional recovery and endogenous neurogenesis in a rat model of MCAO.

## 2. Material and Methods

### 2.1. Preparation of Human MSCs

Human bone marrow-derived mesenchymal stem cells (Catholic MASTER Cells) were obtained from Catholic Institute of Cell Therapy (CIC, Seoul, Korea). Human bone marrow (BM) aspirates were obtained from the iliac crest of healthy donors aged 20 to 55 years after approval by the Institutional Review Board of Seoul St. Mary's Hospital (approval numbers KIRB-00344-009 and KIRB-00362-006). Bone marrow aspirate from each consented donor was collected and sent to the GMP-compliant facility of Catholic Institute of Cell Therapy (Seoul, Korea, http://www.cic.re.kr) for the isolation, expansion, and quality control of human marrow-derived mesenchymal stem cells (hBM-MSCs). The marrow mixture was centrifuged at 4°C, 793 g for 7 minutes to obtain a marrow pellet. After removal of the supernatant, red blood cells were removed by adding and suspending in 10-fold volume of sterile distilled water. Cell pellet obtained by centrifugating the RBC-deprived sample was then suspended in MSC growth medium (Dulbecco's modified Eagle's medium-low glucose (DMEM-LG, PAA, Austria), 20% fetal bovine serum (FBS, PAA, Austria)). They were added to T-75 tissue culture flasks (NUNC, NY, USA), which were placed in CO_2_ incubator to initiate culture. The incubator was maintained at 37°C with 5% CO_2_. The MSC growth medium was used for all cell expansion procedures, unless mentioned otherwise. Media were replaced twice per week. Cells were detached when they reached 70~90% confluence and replated at a density of 5 ~ 8 × 10^3^ cells/cm^2^. Cells were expanded 2 to 4 passages in the GMP-compliant facility. During cell expansion, cells were tested for bacterial sterility, mycoplasma sterility, and endotoxin level (<3 EU/mL). In addition, multidifferentiation potential and cellular surface antigens (CD90/CD73, >95% positive; CD34/CD45, >95% negative) were tested for cells after 4th passage.

A recombinant replication-defective adenovirus (rAd) expressing human BDNF was generated using the AdEasy Vector System (QBioGene) as previously described [21]. To transfect hUCB-MSCs, adenoviruses at a specified MOI were pretreated with protein transduction domain (PTD) [22].

### 2.2. MCAO Model

Rat MCAO was used as the stroke model. Transient MCAO was performed using a previously described method of intraluminal vascular occlusion [23]. Adult male Sprague-Dawley rats weighing 270–300 g were initially anesthetized with 5% isoflurane and maintained under anesthesia with 1.5% isoflurane in a mixture of 70% N_2_O and 30% O_2_ using a face mask. Body temperature (as assessed via rectal measurement) was maintained at 37°C with a heating pad (Panlab S.L., Barocelona, Spain). A 20.0 mm 4–0 surgical Dermalon suture with the tip rounded by heating near a flame was advanced from the right external carotid artery (ECA) into the lumen of the internal carotid artery (ICA) until it blocked the origin of the middle cerebral artery (MCA). Ninety minutes after the MCAO, the animal was reanesthetized with isoflurane and reperfusion was performed by withdrawal of the suture until the tip cleared the lumen of the ECA. All animal protocols were approved by the Institutional Animal Care and Use Committee in School of Medicine, The Catholic University of Korea.

### 2.3. Transplantation of MSCs

Stereotaxic surgery was performed with animals under isoflurane anesthesia 3 days post-MCAO. We transplanted 5 *μ*L suspensions of 5 × 10^5^ cells in PBS into one site ipsilateral (AP: +1.0; ML: +2.2; DV: −5.0) to the lesion. Experiments consisted of three groups (*n* = 69) assigned randomly: administration of PBS alone (control) (*n* = 23), of naive MSCs (*n* = 23), or of MSCs-BDNF (*n* = 23). A microinfusion pump (KD scientific, Holliston, MA) was used to maintain the speed of delivery at 0.5 *μ*L/min. The needle was left in situ for 2 min postinjection, before slow removal. The rats were injected with 5-bromo-2′-deoxyuridine (BrdU, 50 mg/kg; Sigma, St. Louis, MO) on the day of transplantation and daily for 4 days after the transplantation.

### 2.4. Behavior Tests

Behavior tests in animals were performed before the MCAO and at 1, 7, 14, and 28 days after MCAO. A blind tester assessed sensorimotor function using an adhesive-removal method. Adhesive paper dots (12*∅*) were used as tactile stimuli on the wrist of the left forepaw, and animals were observed in a cage. The time, to a maximum of 3 min, necessary for each rat to remove the tape from the forelimb (removal time) was recorded in three trials per day. An accelerating rotarod (Ugo Basile, Italy) evaluation was used to measure rat motor function. The rats were placed on a rotarod cylinder, and the time that the animal remained on the rotarod was measured. The speed was increased from 4 to 40 rpm within 5 min. The trial ended if the animal fell off the rung or gripped the device and spun around for two consecutive revolutions without attempting to walk on the rung. The data are presented as the percentage of the mean duration (three trials) on the rotarod compared with an initial baseline control (before MCAO).

### 2.5. Detection of BDNF In Vitro and In Vivo

Culture supernatants were collected for analysis 48 h after hUCB-MSCs were transfected in vitro at various MOIs (pfu/cell). One week after MCAO, rats were anesthetized with ketamine and xylazine i.p., their brains were removed, and coronal sections (100 mg) from −1.0 to +2.0 mm to bregma in the ischemic hemisphere were dissected on ice and stored −80°C until use. Subsequently, each tissue sample was suspended in an equal weight of homogenate buffer (T-PER Reagent; Thermo Scientific, Rockford, IL) and homogenized using a Precellys 24 homogenizer (Bertin, France). The homogenate was centrifuged (10,000 ×g) for 10 min at 4°C, and the supernatant was collected for analysis. Commercial BDNF enzyme-linked immunosorbent assay (ELISA) kits (Chemicon, Temecula, CA) were used to quantify the concentration of BDNF in each sample.

### 2.6. TTC Staining and Quantitative Analysis of Infarct Volume

Rats were anesthetized i.p. with ketamine and xylazine 2 weeks after MCAO. The brains were removed carefully and dissected into coronal sections (2 mm thick). The fresh brain slices were immersed in a 2% solution of 2,3,5-triphenyltetrazolium chloride (TTC; Sigma) in PBS at 37°C for 30 min. The cross-sectional area of each slice of infarcted brain was measured using image analysis software (MetaMorph, PA, USA). The total infarct volume for each slice was calculated by summation of infarcted areas of all brain slices. The corrected infarct volume (CIV) was calculated as

(1) $$\begin{array}{c} \text{CIV}=\left[\text{LT}-\left(\text{RT}-\text{RI}\right)\right]\times d, \end{array}$$

where LT is the area of the left hemisphere, RT is the area of the right hemisphere, RI is the infarcted area, and *d* is the slice thickness (2 mm) [24].

### 2.7. Immunohistochemical Staining

Seven and 28 days after stroke, experimental models were sacrificed for immunohistochemistry. Animals were intracardially perfused with PBS and then fixed with 4% paraformaldehyde. The excised brains were postfixed overnight and then equilibrated in 30% sucrose solution for 1 day. Fixed brains were embedded, snap frozen in liquid nitrogen, and stored at −80°C until use. Tissues were cryosectioned to a 10 *μ*m thickness. To identify the BDNF level, the tissues were probed with an anti-BDNF antibody (Chemicon). To evaluate the endogenous cell proliferation and endogenous neurogenesis, tissues were double stained with anti-BrdU (Serotec, Oxford, UK), antidoublecortin (DCX; Chemicon), and anti-NeuN (Chemicon) antibodies. The immunoreactivity was visualized using Cy2-conjugated anti-IgG (Jackson, West Grove, PA) or Alexa Fluor 488- or 633-conjugated anti-IgG (Invitrogen) secondary antibodies. Confocal images were obtained using a Zeiss laser scanning confocal microscope (LSM 510 Meta, Carl Zeiss) and Zeiss software.

At 14 days after stroke, coronal cryosections (10 *μ*m thick) from animals of each group were stained using the terminal deoxyribonucleotidyl transferase-mediated dUTP nick end labeling (TUNEL) assay kit (Roche, Mannheim, Germany) and developed using Cy2-conjugated streptavidin (Jackson ImmunoResearch Laboratories). Sections were counterstained with 4′,6-diamidino-2-phenylindole (DAPI; Sigma). The total number of TUNEL-positive cells and DAPI counterstaining-positive cells was counted individually using fluorescence microscopy in three slides from each brain, with each slide containing three fields from the ischemic boundary zone and one field from the ischemic core.

### 2.8. Statistical Analysis

All data are expressed as mean ± SD. Statistical differences between test conditions were determined using Student's *t*-test or one-way ANOVA with Tukey's *post hoc* test. Significance was set at *P* < 0.05.

## 3. Results

### 3.1. Detection of Immunoreactive Human BDNF and Quantitative Analysis In Vitro and In Vivo

**Figure 1:**
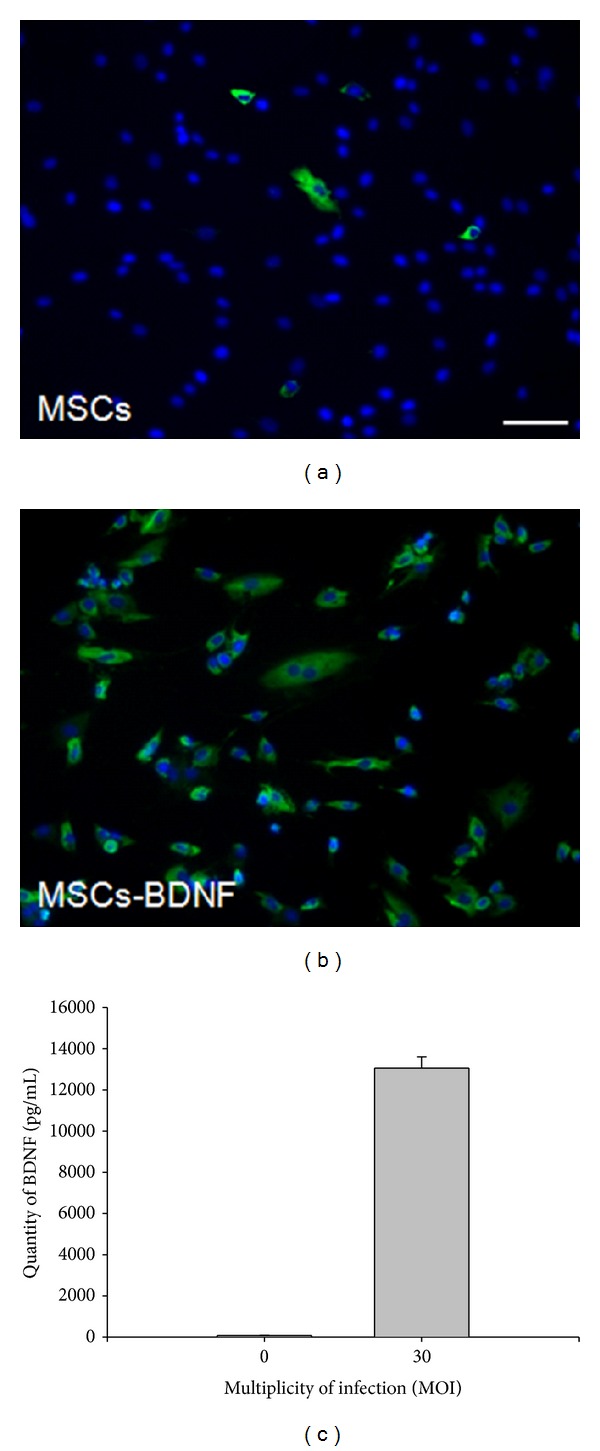
BDNF immunostaining in primary MSCs (a) and MSCs-BDNF (b) (scale bar: 50 *μ*m). (c) Secreted BDNF levels in the supernatant of MSCs transfected with Ad-BDNF (MSCs-BDNF) at an MOI of 30.

The levels of BDNF in the supernatant of cultured MSCs and MSCs transfected with adenoviruses expressing human BDNF (MSCs-BDNF) with PTD at an MOI of 3, 10, and 30 pfu/cell are shown in Figure 1. Transfected MSCs secreted BDNF at a rate of 643.63, 7930.90, and 13229.09 pg/mL/48 h when infected at an MOI of 3, 10, and 30, respectively. Nontransfected MSCs also produced BDNF protein (72.72 pg/mL/48 h). The level of BDNF production from MSCs-BDNF transfected at an MOI of 30 was 180-fold greater than that of noninfected MSCs.

**Figure 2:**
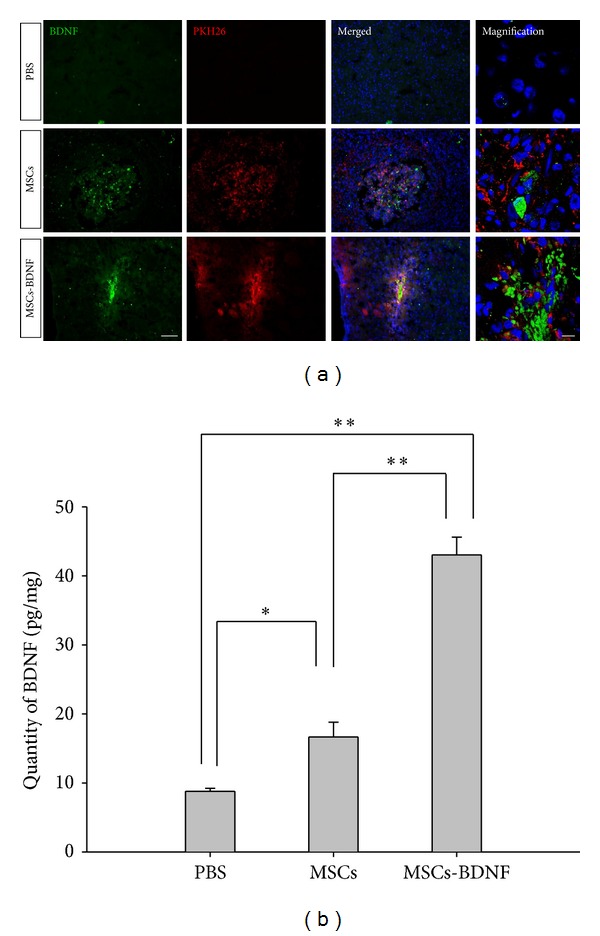
In vivo levels of BDNF assayed using immunostaining and ELISA. Seven days after MCAO, sections were obtained through the SVZ of the ischemic hemisphere ((a), MSCs and MSCs-BDNF: red; BDNF: green; scale bar: 100 *μ*m, magnified image: 10 *μ*m). The level of BDNF increased significantly in the ischemic hemisphere of animals in the MSCs and MSCs-BDNF groups compared with PBS-treated rats (b) (*t* test, *P < 0.05 and **P < 0.01).

BDNF protein was diffusely overexpressed in the ipsilateral brain in the MSCs-BDNF group (Figure 2). A high level of BDNF expression in the MSCs-BDNF group was detected in the ipsilateral striatum 7 days after MCAO. However, virtually no BDNF expression was detected on the contralateral hemisphere (data not shown).

The levels of BDNF in brain tissues were measured using sandwich ELISA 7 days after MCAO. BDNF levels increased significantly in the ischemic hemisphere of the MSCs group (16.66 ± 2.14 pg/mg) compared with the PBS group (8.78 ± 0.42 pg/mg). In the MSCs-BDNF group, BDNF increased in the ischemic hemisphere (43.03 ± 2.57 pg/mg) compared with the PBS and MSCs groups (Figure 2(b)).

### 3.2. Therapeutic Effects of MSCs-BDNF in the Rat Stroke Model

Numerous studies have reported that MSC treatment enhances poststroke recovery [1–3]. Similarly, systemic BDNF treatment has been tailored to enhance poststroke recovery, similar to sensorimotor function [15]. To evaluate the therapeutic effects of MSCs-BDNF, 5 × 10^5^ MSCs or MSCs-BDNF were transplanted into the ischemic boundary zone 3 days after MCAO.

**Figure 3:**
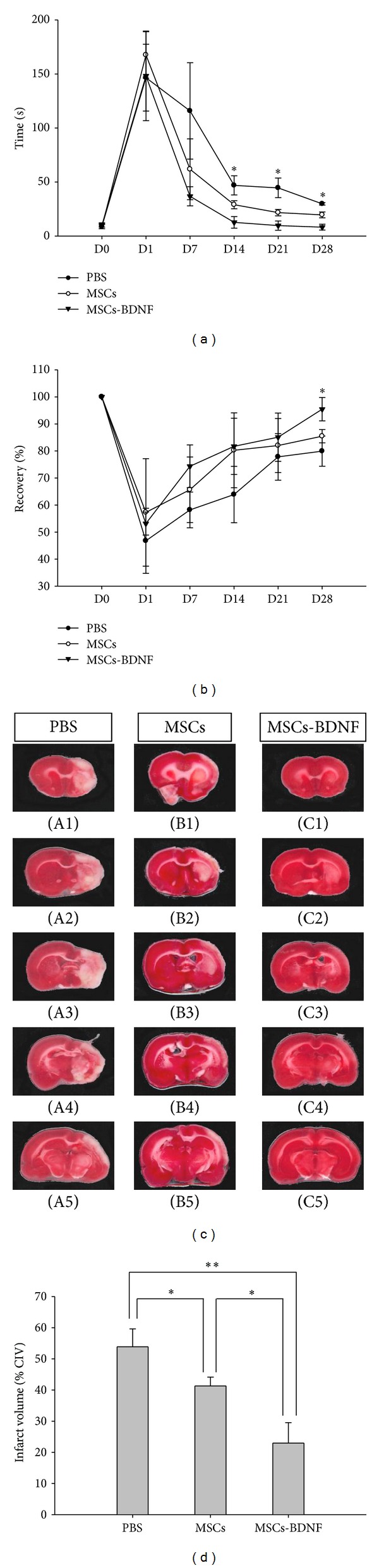
Transplantation of MSCs-BDNF improved functional outcome after ischemia. (a) The MSCs and MSCs-BDNF groups exhibited better sensorimotor function compared with the PBS group (*n* = 5, each group). The MSCs-BDNF group exhibited the fastest recovery compared with the other groups (*P < 0.05). (b) The MSCs-BDNF group had better running function compared with the other groups at 28 days after MCAO (*P < 0.05). (c) Brain slices were stained with TTC to visualize lesions (*n* = 3, each group). (d) 14 days after MCAO, significant differences of %CIV were detected in each group (*P < 0.05 and **P < 0.01).

The results of the adhesive-removal test were statistically different between the PBS-treated group (control) and the cell-treated groups (MSCs and MSCs-BDNF) (Figure 3(a)). One day after cell transplantation, there were no significant differences among the three groups. However, 14 days after MCAO, significant differences were evident: MSCs-BDNF (12.66 ± 5.36 s), MSCs (29 ± 3.75 s), and PBS (46.88 ± 8.87 s) (*P* < 0.05). Significant differences persisted on days 21 and 28. The MSCs-BDNF group showed the fastest recovery, and the PBS group showed the slowest improvement (*P* < 0.05). The results of the rotarod stress test performed at the same time did not reveal a significant difference until 21 days after MCAO. However, 28 days after MCAO, the MSCs-BDNF group displayed a significant improvement (95.4 ± 4.33%) compared with the PBS group (79.9 ± 5.58%) and the MSCs group (85.4 ± 2.47%) (*P* < 0.05) (Figure 3(b)).

We compared the infarction areas in coronal sections from animals of the PBS, MSCs, and MSCs-BDNF groups on day 14 (Figure 3(c)). TTC staining was used to assess lesion volume as a percentage of contralateral hemispheric volume. 14 days after MCAO, significant differences of %CIV were detected in the MSCs-BDNF group compared with the MSCs and PBS groups (22.94 ± 8.30% versus 41.28 ± 2.85% versus 53.86 ± 8.60%; *P < 0.05, **P < 0.01).

### 3.3. Enhanced Neurogenesis by MSCs-BDNF

Data obtained in the present study were consistent with the notion that MSCs promote endogenous neurogenesis in experimental stroke models. We investigated whether transplantation of MSCs-BDNF enhanced neurogenesis to a greater extent than transplantation of MSCs alone. We found immunoreactivity for the cells proliferation indicator BrdU and/or the migrating neuroblast marker DCX in the SVZ.

**Figure 4:**
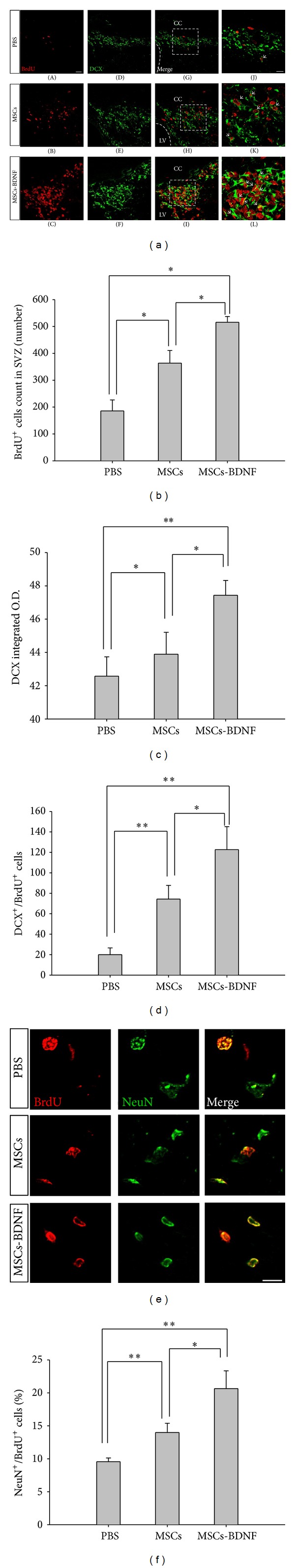
MSCs-BDNF increased endogenous neurogenesis in the ischemic stroke model. (a) Area of the SVZ exhibiting migrating neuroblasts (DCX; green). Coexpression of BrdU (red) and DCX (green) in cells of the SVZ. Arrows indicate double-positive (BrdU/DCX) cells. MSCs-BDNF led to an increase in the number of new neuronal cells after cell transplantation (scale bar: (A)–(I); 20 *μ*m, (J)–(L); 10 *μ*m). (b) Significant increase in the number of BrdU^+^ cells in the SVZ of the MSCs and MSCs-BDNF groups compared with the PBS group (*P < 0.05). (c) Quantification of DCX immunoreactivity (*P < 0.05 and **P < 0.01). (d) Quantification of DCX/BrdU double-positive cells in the SVZ (*P < 0.05 and **P < 0.01). (e) Coexpression of BrdU (red) and NeuN (mature neuronal marker; green) in the ipsilateral striatum at 4 weeks after MCAO (scale bar: 10 *μ*m). (f) Quantification of NeuN/BrdU double-positive immunoreactivity (*P < 0.05 and **P < 0.01).

Seven days after MCAO, a significant increase in the number of BrdU^+^ cells in the SVZ of the MSCs group and the MSCs-BDNF groups was evident, compared with the PBS group (363.66 ± 47.00 versus 515.66 ± 21.51 versus 185.66 ± 40.91, *P* < 0.05) (Figure 4(b)). The intensity of SVZ DCX^+^ immunoreactivity indicated that the MSCs-BDNF group was much thicker and contained more DCX^+^ cells than that of the MSCs and PBS groups. Integrated optical density measurements of DCX^+^ cells showed that the MSCs-BDNF group possessed the highest positive immunoreactivity (PBS: 42.57 ± 1.16, MSCs: 43.89 ± 1.31, and MSCs-BDNF: 47.435 ± 0.88, PBS versus MSCs: *P* < 0.05, PBS versus MSCs-BDNF: *P* < 0.01, and MSCs versus MSCs-BDNF: *P* < 0.05) (Figure 4(c)). Moreover, the number of DCX/BrdU double-positive cells increased robustly after stem cell transplantation (PBS: 20 ± 6.55, MSCs: 74.33 ± 13.31, and MSCs-BDNF: 122.66 ± 22.50, PBS versus MSCs: *P* < 0.01, PBS versus MSCs-BDNF: *P* < 0.01, MSCs versus MSCs-BDNF: *P* < 0.05) (Figure 4(d)).

Four weeks after MCAO, we determined the neuronal fraction among the new cells (BrdU^+^/NeuN^+^) using microscopy. Labeling with the mature neuron marker NeuN showed that many cells were positive near the IBZ in animals of the MSCs-BDNF- and MSCs-transplanted groups (Figure 4(e)). Compared with all other groups, the MSCs-BDNF group displayed the highest prevalence of positive double-labeled cells (PBS: 9.85 ± 0.29%, MSCs: 13.44 ± 1.48%, and MSCs-BDNF: 20.65 ± 3.80%; PBS versus MSCs: *P* < 0.01, PBS versus MSCs-BDNF: *P* < 0.01, and MSCs versus MSCs-BDNF: *P* < 0.05) (Figure 4(f)).

### 3.4. Antiapoptosis

**Figure 5:**
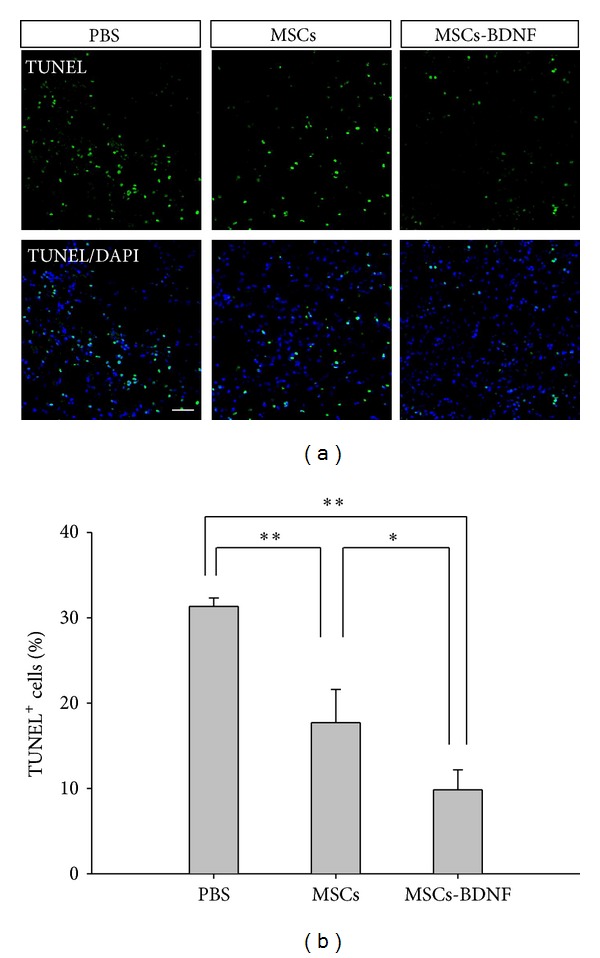
Apoptotic cells identified using TUNEL staining. (a) Fewer TUNEL-positive cells were detected in rats transplanted with MSCs-BDNF compared with the other groups (MSCs or PBS) (TUNEL: green; DAPI: blue; scale bar: 50 *μ*m). (b) The percentage of TUNEL-positive cells in the ischemic lesion was significantly reduced in the MSCs-BDNF group compared with the other groups (*P < 0.05 and **P < 0.01).

Using TUNEL staining (Figure 5), apoptotic cells with green fluorescence were counted in the ischemic boundary zone and ischemic core 2 weeks after the ischemic injury. In addition, cells with blue fluorescence (DAPI staining) were counted in the same area. In this area, we did not accept donor MSCs (red fluorescence); therefore, the cells counted seemed to be host derived. The percentage of apoptotic host cells was significantly decreased in the MSCs-BDNF group (9.85 ± 2.33%) compared with the PBS (31.32 ± 0.99%, *P* < 0.01) and MSCs (17.71 ± 3.88%, *P* < 0.05) groups. The difference between the latter two groups was significant (*P* < 0.01).

## 4. Discussion

The present study demonstrated that transplantation of either MSCs-BDNF or MSCs enhanced functional recovery, which correlated with enhanced proliferation of endogenous NSCs in the SVZ and in the neuronal maturation of newborn neuroblasts. The recovery was associated with reduction in infarct volume, protection from apoptotic cell death, improvement in behavioral performance, increased BDNF levels in the infarcted cerebral hemisphere, and enhanced endogenous neurogenesis.

MSCs can be isolated from many different adult tissues. Numerous studies have reported beneficial effects of MSCs in tissue repair and regeneration [25]. MSCs secrete a variety of bioactive substances such as neurotrophins (e.g., NGF and BDNF), interleukins, macrophage colony-stimulating factor, and stem-cell factors [26]. In addition, MSCs home to and engraft into injured tissues and modulate the inflammatory response via synergistic downregulation of proinflammatory cytokines and upregulation of both prosurvival and anti-inflammatory factors [25]. Intracranial infusion of BDNF significantly reduces infarct volume after induction of cerebral ischemia [27], and intravenous infusion of BDNF enhances functional recovery and endogenous neurogenesis [15], suggesting that BDNF may protect cells from ischemic injury. However, usually recombinant proteins activation is short in vivo. The persistence of transgene expression in MSCs transduced with adenovirus was analyzed in our previous study. We found that the expression from MSCs inoculated into experimental mice persisted for 14 days [22]. Therefore we used BDNF gene expressed MSCs in this study. To investigate the effect of BDNF on the neuroprotective and endogenous neurogenesis action of transplanted MSCs in MCAO, we compared the effects of MSCs with those of MSCs-BDNF.

The BDNF gene was introduced into MSCs using a recombinant replication-deficient adenoviral vector encoding *BDNF* (Ad-BDNF) with PTD. Elevated secretion of BDNF protein in these cells compared with nontransgenic MSCs was confirmed in vitro using ELISA. BDNF production by MSCs-BDNF was 180-fold greater than that observed in uninfected MSCs. BDNF levels assayed by ELISA in the ischemic brain lesion increased after the transplantation of either MSCs or MSCs-BDNF. However, levels were significantly higher in the latter, suggesting that MSCs-BDNF can maintain high levels of BDNF during the critical postischemic period, and that this elevated BDNF secretion contributes to the enhanced neuroprotection and endogenous neurogenesis.

The present study showed improvement in behavioral performance and reduction in infarct volume. We performed an adhesive-removal test and a rotarod stress test. These can be used to demonstrate behavioral therapeutic effects on MCAO injured rats (Figures 3(a) and 3(b)). We performed statistical analysis using one-way ANOVA with Tukey's *post hoc* test. We measured infarction volume using the TTC staining method. TTC measures the mitochondrial activity of all cells. TTC staining results in viable tissues appearing in a “brick-red,” as the tetrazolium salts react with dehydrogenates in the cells, whereas infarcted tissues stain a pale white, as they lack the enzymes with which the TTC reacts. However, some cells that infiltrate the infarct lesion can be stained. We measured infarct size at 14 days after stroke. It makes some concerned. This study showed therapeutic effects at least 14 days after stroke. Thus, we decided to use 14 days after stroke as the time point for analysis. We compared the infarction areas among the groups. The MSCs-BDNF group exhibited the best recovery (Figure 3(d)).

Neurogenesis persists in the adult brain, where it may contribute to repair and recovery after injury [8, 28]. Hence, multipotent cells located in the hippocampus hilus and SVZ of the lateral ventricle manifest as increased proliferation and migration in pathological situations. Moreover, progenitor cells such as migratory neuroblasts (doublecortin; DCX) that migrate through the rostral migratory stream from the SVZ to the olfactory bulb can be triggered to differentiate into neurons [28, 29]. After ischemic injury, the SVZ expands [30, 31] and proliferation of NSCs is accelerated during the first 2 weeks compared with the normal brain [6]. The newly generated DCX^+^ cells migrate toward the ischemic territory [31].

The results of the present study are consistent with observations that MSCs promote endogenous neurogenesis in stroke models. Upregulation of BDNF levels has been associated with recruitment of neural progenitors of the forebrain [32]. We investigated whether the transplantation of MSCs-BDNF enhances endogenous neurogenesis to a greater extent compared with transplantation of MSCs alone. Transplantation of MSCs-BDNF yielded the highest number of BrdU^+^ and DCX^+^ cells in the SVZ 7 days after MCAO, compared with rats transplanted only with MSCs or PBS (Figure 4). These results indicate that the BrdU^+^ and DCX^+^ cells in the SVZ are derived from the host NSCs, and that the density of these cells increases in response to the BDNF secreted by MSCs-BDNF. In addition, cells that were positive for both NeuN and BrdU in the ipsilateral lesion were evident 4 weeks after MCAO (Figures 4(e) and 4(f)). BrdU is commonly used for the detection of proliferating cells in living tissues. BrdU was administered until 4 days after cell transplantation. BrdU^+^ cells only proliferated after cell transplantation. These findings are consistent with the view that the therapeutic effects of MSCs-BDNF are associated with the acceleration of proliferation maturation of NSCs resident in the SVZ.

## 5. Conclusions

In conclusion, the transplantation of either MSCs or MSCs-BDNF (transfected with the BDNF gene using an adenovirus vector and PTD) resulted in reduced ischemic damage and improved function in a rat MCAO model. Transplantation of MSCs-BDNF was most efficacious. The transplantation of MSCs or MSCs-BDNF affected the ischemic brain largely by secreting neurotrophic factors. The transplantation of MSCs-BDNF can enhance the protective effect by preventing apoptosis in the ischemic brain and by promoting the proliferation of endogenous stem cells.
